
JOURNAL INFORMATION
==============================
NLM Title Abbreviation: J Clin Lab Anal
Journal ID: J Clin Lab Anal
NLM Journal ID: 8801384
Journal ID: JCLA

Journal of Clinical Laboratory Analysis

ISSN: 0887-8013
EISSN: 1098-2825
Publisher: Wiley

ARTICLE INFORMATION
==============================
PMCID: PMC11474506
DOI: 10.1002/jcla.25014
Article ID: JCLA25014
Article version: 1
Subjects: Issue Information, Issue Information

Issue Information

Electronic publication date: 2024 Jan 31
Publication date: 2024 Jan
Volume: 38
Issue: 1-2
Electronic Location ID: e25014
Copyright: © 2024 The Authors. Journal of Clinical Laboratory Analysis published by Wiley Periodicals LLC.
License: This is an open access article under the terms of the http://creativecommons.org/licenses/by/4.0/ License, which permits use, distribution and reproduction in any medium, provided the original work is properly cited.
License URL: https://creativecommons.org/licenses/by/4.0/

Figure count: 0
Table count: 0
Page count: 2

source-schema-version-number: 2.0
cover-date: January 2024
details-of-publishers-convertor: Converter:WILEY_ML3GV2_TO_JATSPMC version:6.4.9 mode:remove_FC converted:15.10.2024

==============================

Volume 38, Issue 1–2 2024

### JCLA Journal of Clinical Laboratory Analysis

Open Access

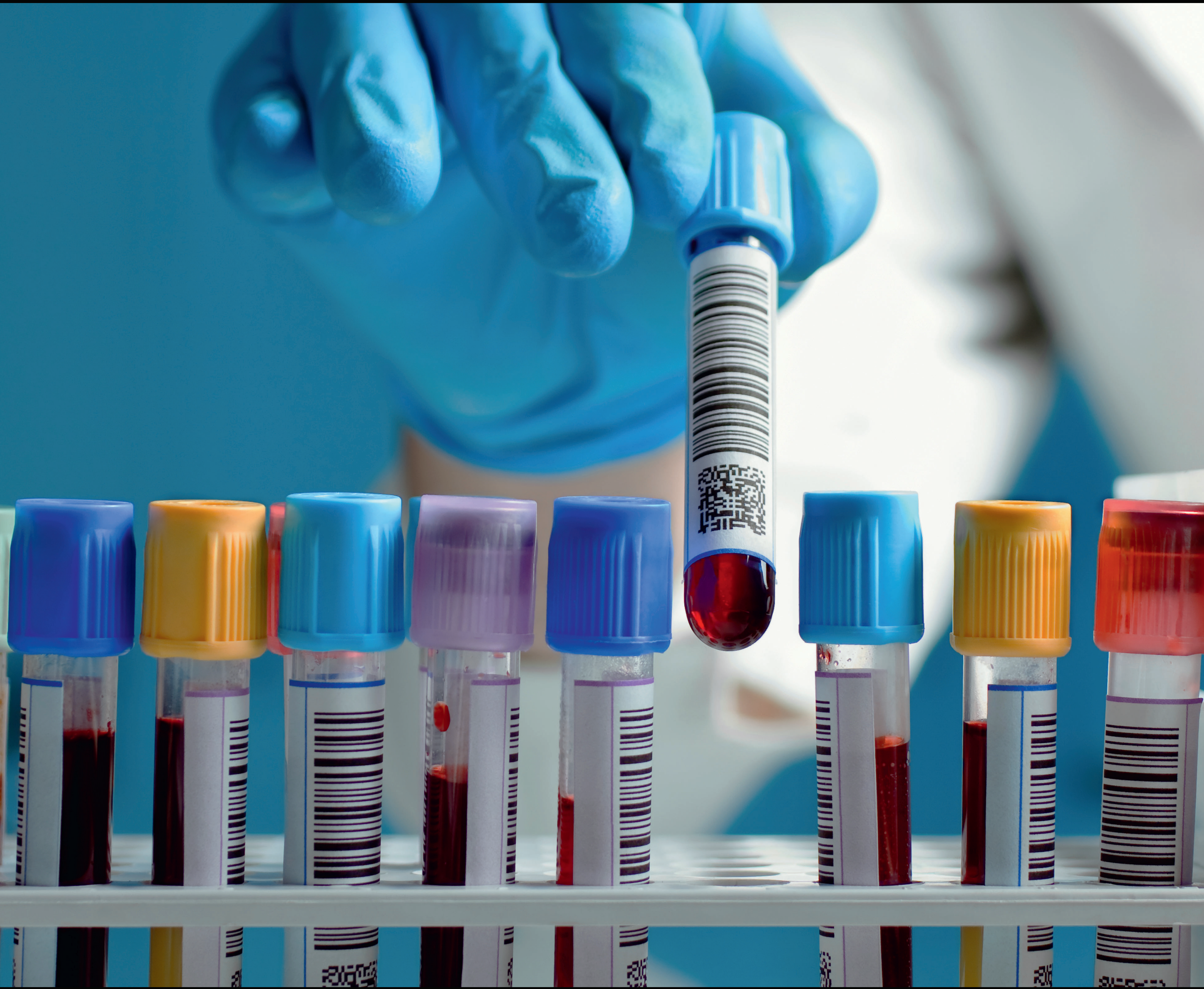

WILEY

### Journal of Clinical Laboratory Analysis

Incorporating *Diagnostic and Clinical Immunology*

### Editor

**Rong Fu,** General Hospital, Tianjin Medical University, Tianjin, China

### Associate Editors

**Xifeng Dong**
General Hospital of Tianjin Medical University, Tianjin, China

**Chunfang Gao**
Yueyang Hospital of Integrated Traditional Chinese and Western Medicine, Shanghai University of Traditional Chinese Medicine, Shanghai, China

**Daguang Wang**
The First Hospital of Jilin University, Changchun, China

**Le Yu**
Southern Medical University, Guangzhou, China

**Lin Zhou**
Shanghai Changzheng Hospital, Shanghai, China

### Editorial Board

**Jun Chen**
Tianjin Medical University General Hospital, Tianjin, China

**Amitava Dasgupta**
University of Texas, Texas, US

**Laurence M. Demers**
The Pennsylvania State University, Pennsylvania, US

**Caigan Du**
The University of British Columbia, Vancouver, Canada

**Andrea Ferreira-Gonzalez**
Virginia Commonwealth University, Virginia, US

**Feng Jiang**
Obstetrics and Gynecology Hospital of Fudan University, Shanghai, China

**Rongcai Jiang**
Tianjin Medical University General Hospital, Tianjin, China

**Mark A. Jandreski**
Siemens Healthcare Diagnostics, Inc., New York, US

**Jinhui Liu**
The First Affiliated Hospital of Nanjing Medical University, Nanjing, China

**Ming Liu**
Tianjin Medical University General Hospital, Tianjin, China

**Junxuan (Johnny) Lu**
Penn State College of Medicine, Pennsylvania, US

**Qishui Ou**
Fujian Medical University, Fujian, China

**Roger S. Riley**
Virginia Commonwealth University, Virginia, US

**Jeffrey Schlom**
Center for Cancer Research, National Cancer Institute, Maryland, US

**Xianrang Song**
Shandong Cancer Hospital, Shandong, China

**Gabriel Virella**
Medical University of South Carolina, South Carolina, US

**Wenrong Xu**
Jiangsu University, Jiangsu, China

**Jun-Jun Wang**
The General Hospital of Eastern Theater command, Jiangsu, China

**Xianglin Wu**
Mayo Clinic, Minnesota, US

**Jun Zhang**
Zhejiang University, Zhejiang, China

**Jun Zhou**
Nankai University, Tianjin, China

**Disclaimer:** The Publisher and Editors cannot be held responsible for any errors in or any consequences arising from the use of information contained in this journal. The views and opinions expressed do not necessarily reflect those of the Publisher and Editors, neither does the publication of advertisements constitute any endorsement by the Publisher, Editors, or Authors of the products advertised.

**Corporate Responsibility & ESG:** ESG (Environmental, Social, Governance) is essential to Wiley's mission of unlocking human potential. For over two centuries, Wiley has a history of helping the world's researchers, learners, innovators, and leaders achieve their goals and solve the world's most important challenges. We take our role as a corporate citizen seriously and are guided by transparency, accountability, and industry best practices. Through intentional climate action and strategic social impact, ESG advances Wiley's commitment to sustainability, positive impact, and leading global change. Follow our progress at https://www.wiley.com/en-us/corporate-responsibility.

**Statement on Research4Life:** Wiley is a founding member of the UN-backed HINARI, AGORA, and OARE initiatives. They are now collectively known as HYPERLINK "https://www.research4life.org/"Research4Life, making online scientific content available free or at nominal cost to researchers in developing countries and enabling more researchers to publish open access by providing publisher backed waivers and discounts. Please visit Wiley's Content Access – Corporate Citizenship site: https://www.wiley.com/en-us/corporate-responsibility.

**Abstracting and Indexing Services:** The Journal is indexed by Science Citation Index, MEDLINE, and CINAHL. For a complete list of A&I services please visit the journal homepage at www.wileyonlinelibrary.com/journal/jcla.

ISSN 1098-2825 (Online)
View this journal online at www.wileyonlinelibrary.com/journal/jcla

Cover image © angellodeco/Shutterstock
